# The influence of population characteristics on variation in general practice based morbidity estimations

**DOI:** 10.1186/1471-2458-11-887

**Published:** 2011-11-24

**Authors:** C van den Dungen, N Hoeymans, HC Boshuizen, M van den Akker, MCJ Biermans, K van Boven, HJ Brouwer, RA Verheij, MWM de Waal, FG Schellevis, GP Westert

**Affiliations:** 1 National Institute for Public Health and the Environment (RIVM), PO Box 1, 3720 BA, Bilthoven, the Netherlands; 2 Department Tranzo, Faculty of Social and Behavioural Sciences, Tilburg University, Tilburg, the Netherlands; 3 Department of General Practice, School for Public Health and Primary Care (Caphri), Maastricht University, Maastricht, the Netherlands; 4 Department of Primary and Community Care, Radboud University Nijmegen Medical Centre, Nijmegen, the Netherlands; 5 General Practitioner, Franeker, the Netherlands; 6 Department of General Practice, Academic Medical Centre/University of Amsterdam, Amsterdam, the Netherlands; 7 Netherlands Institute for Health Services Research (NIVEL), Utrecht, the Netherlands; 8 Department of Public Health and Primary Care, Leiden University Medical Centre, Leiden, the Netherlands; 9 Department of General Practice/EMGO Institute for health and care research, VU University Medical Centre, Amsterdam, the Netherlands; 10 Scientific Institute for Quality of Healthcare (IQ healthcare), Radboud University Nijmegen Medical Centre, Nijmegen, the Netherlands; 11 Department of General Practice, Katholieke Universiteit, Leuven, Belgium

**Keywords:** Family practice, Incidence, Medical records, Population characteristics, Public health, Prevalence

## Abstract

**Background:**

General practice based registration networks (GPRNs) provide information on morbidity rates in the population. Morbidity rate estimates from different GPRNs, however, reveal considerable, unexplained differences. We studied the range and variation in morbidity estimates, as well as the extent to which the differences in morbidity rates between general practices and networks change if socio-demographic characteristics of the listed patient populations are taken into account.

**Methods:**

The variation in incidence and prevalence rates of thirteen diseases among six Dutch GPRNs and the influence of age, gender, socio economic status (SES), urbanization level, and ethnicity are analyzed using multilevel logistic regression analysis. Results are expressed in median odds ratios (MOR).

**Results:**

We observed large differences in morbidity rate estimates both on the level of general practices as on the level of networks. The differences in SES, urbanization level and ethnicity distribution among the networks' practice populations are substantial. The variation in morbidity rate estimates among networks did not decrease after adjusting for these socio-demographic characteristics.

**Conclusion:**

Socio-demographic characteristics of populations do not explain the differences in morbidity estimations among GPRNs.

## Background

Policy makers need valid epidemiological information about the incidence and prevalence rates of diseases in the population to formulate public health policy. Every four years, the Dutch Public Health Status and Forecasts Report presents an overview of the population's health status using key public health indicators such as (healthy) life expectancy, morbidity rates and health determinants [1,2]. In this report general practice based data are used to estimate the population's morbidity in terms of incidence and prevalence rates of many diseases.

Using data generated by general practice registration networks (GPRNs) to estimate morbidity has many advantages, especially in countries with a strong primary care system, like the United Kingdom and the Netherlands [3-5]. In these countries, all non-institutionalized residents are listed with a single general practitioner (GP), which makes a precise determination of the population at risk possible.

GPRNs put a lot of effort in building a reliable database. GPs, who belong to the same GPRN, are expected to use uniform recording methods and classification systems to record diseases. Furthermore, GPRNs systematically check the data to assure quality. Still, GPRNs differ from each other on several aspects. For example, there are GPRNs that include all morbidity presented in general practice, 'episode based' registries, while others only record chronic or very serious conditions into their database, also called 'problem based' registries [4].

In a previous paper, we identified possible explanations for differences in morbidity rates among Dutch GPRNs and categorized them into four types of factors, health care system, methodology, practice/practitioner characteristics and patient characteristics. Until now, the contribution and mechanisms of these factors on the differences in morbidity estimation among GPRNs are not fully understood [3,4]. To improve the usability of GPRN data for morbidity estimations of the total national population these aspects need to be investigated.

In this paper we investigate the effect of differences in patient characteristics on variation in morbidity estimations among GPRNs. Age, gender, socio-economic status (SES), urbanization level and ethnicity affect the probability to be diagnosed with a certain disease. For example, 65 percent of the people in low socio-economic class is chronically ill compared to nearly 40 percent of the people in the highest socio-economic class [1]. There is reason to believe that the distribution of population characteristics varies among GPRNs, because some networks only operate in urban areas, while others operate in both urban and rural areas [4]. Furthermore, most networks operate in a specific region, while immigrants are not equally spread across the Netherlands [6].

Before investigating the effect of socio-demographic characteristics on the variation in morbidity among GPRNs, we studied the variation between networks and practices. We assume that for diseases with more ambiguous diagnostic criteria (e.g. depression) the variation among networks and among practices is larger than for diseases with clear diagnostic criteria (e.g. diabetes mellitus) [7]. For diseases with disease-free periods (e.g. dermatitis, depression), we expect more variation in prevalence rates than in incidence rates [8,9]. These differences result from difficulties in determining the ending of an episode in the registration. An episode starts when a GP records information about a patient's health, from contact with the patient or from information about the patient's condition from other health care providers, in the patient's medical record. On the other hand, a GP does not receive information when a disorder is cured [10,11].

In summary, the goal of this paper is to study the variation among general practices and networks in incidence and prevalence rates of a selection of diseases. To gain more insight in possible explanations for these differences in morbidity rates, we investigate the influence of population characteristics. We hypothesize that adjusting for differences in age, gender, SES, urbanization level, and ethnicity among networks will reduce the variation among networks and therefore partly explain the differences in morbidity estimations among GPRNs.

## Methods

### Databases

We used 'episode based' data, which include information about all contacts for a specific health problem of an individual patient. Episodes are defined as the period between the first presentation of a health problem in general practice until the last recorded contact for the same health problem or disease. Episodes contain the coded information about diagnosis, referrals, interventions and prescribed medication [10].

We used data from six Dutch GPRNs, who were able to supply episode based data; the Continuous Morbidity Registration Nijmegen (CMR-N), the General Practice Network Academic Medical Centre (GP-net-AMC) the Netherlands Information Network of General Practice (LINH), the Registration Network of General Practitioners Associated with Leiden University (RNUH-LEO), the Study of Medical Information and Lifestyle in Eindhoven (SMILE) and the Transition project (Trans). Details of these GPRNs and other Dutch databases can be found elsewhere [4].

### Using the data

We performed an observational study without any interventions. In the Netherlands, no approval is necessary from an ethical committee for analyzing data from general practice registration networks. The data are not openly available, permission to use the data is granted by RNUH-LEO, SMILE, Transition project, LINH steering committee, HAG-net-AMC steering committee and the chair of CMR-N.

### Selection of diseases

We selected the diseases on the basis of three criteria. First, the expected occurrence of the disorder in the general Dutch population should be at least 3 per 1000 per year, with a preference for the more common diseases [7]. Second, we aimed to represent all ICD classification chapters to obtain a broad spectrum of diseases (chronic and acute illnesses, psychological and somatic diagnoses, illnesses of different organ systems). Third, we selected a variation of diseases to include a variation of diseases which mainly occur in specific groups of people (young, old people, women, men). Twelve diseases were selected; gastrointestinal tract infections, diabetes mellitus, depression, anxiety disorders, stroke, coronary heart disease (CHD), chronic obstructive pulmonary disease (COPD), asthma, urinary tract infection, dermatitis, osteoarthritis and neck and back problems. Shingles or herpes zoster was added as 13^th ^disease because of its consistent occurrence in the population. Fleming and colleagues demonstrated that the incidence rates of herpes zoster can be used as an indicator of accurate population estimates and it might be used as a indicator of recording quality [12].

### Incidence and prevalence rates

In general, GPs record diagnoses according to the International Classification of Primary Care (ICPC) [13], only one GPRN uses the so-called E-list codes [14,15]. To obtain comparable morbidity rates some codes were combined to determine incidence and prevalence rates. Different codes for neck and back problems are, for example, combined into one disease category. The GPs of all GPRNs are trained to use the classification system properly.

In this study, we used data recorded in 2007. To determine incidence rates we counted all patients with a new episode of a certain disease in the period from January 1 2007 to December 31 2007 per 1, 000 listed patients. The incidence of chronic diseases represents the number of patients that have been diagnosed with the disease for the first time. The incidence figures of acute or recurring illnesses represent the number of patients that at least had one new episode of the disease in 2007. Prevalence rates were calculated by counting the number of patients with a new or an existing episode of a specific disease in 2007 per 1000 listed patients. Incidence rates were calculated for all thirteen diseases; prevalence rates were only calculated for the 10 chronic or recurring diseases. The epidemiological denominator was measured by counting all listed patients adjusted for the number of days a person was registered in the general practice (in case of moving from or to the practice, death or new-borns) in 2007. One GPRN (HAG-net-AMC) had only prevalence data available.

### Socio-demographic characteristics

We analyzed the effect of age, gender, SES, urbanization level and ethnicity. Age (in years) and gender were derived from the central database of the GPRN. SES, urbanization level and ethnicity were determined by proxy using 4-digit postal codes of the patients' home address (the population size is about 4, 000 per postal code area) [16]. The SES score was developed by Knol and colleagues, who estimated SES using principal-component analysis on the basis of different factors indicating socio-economic position, such as average income per household, percentage low income households, percentage unemployed, and percentage households with a low educational level. These indicators are commonly used to determine SES and contribute to a fair estimation of the SES of the population a particular area. The results of this analysis were available on the website of the Netherlands Institute for Social Research (SCP) [17]. The values were divided into quintiles, but to retain the power in our analyses we recoded SES into three categories (1-2 = high, 3 = medium, 4-5 = low SES). Following common practice, urbanization level and ethnicity were derived from Statistics Netherlands [16]. Urbanization level was analysed in three categories; 'very urban', 'urban' and 'rural', based on the total number of addresses in one postal code. Ethnicity was based on the percentage non-western immigrants in a postal code area according to the definition of Statistics Netherlands. To be classified as a non-western immigrant a person or at least one of his/her parents must be born in a non-western country (Turkey, all countries in Africa, countries in Asia or the South-America, except of Netherlands East Indies and Japan). We distinguished four categories: people living in neighbourhoods with almost no (0 < 10%), some (10 < 50%), many (50 < 70%) or most (≥70%) persons from non western origin. This represents the probability that a person is from non-western origin.

### Analyses

Descriptive analyses were applied to get insight into the frequency and distribution of socio-demographic characteristics of the listed patient population of GPRNs. To explore the differences in morbidity rate estimates among GPRNs multilevel logistic regression analysis was used, distinguishing three levels (patient, practice, and network). We used random intercepts on network and practice level to determine the unexplained variation among GPRNs and practices. The differences in morbidity estimations among GPRNs were analyzed by calculating the corresponding median odds ratio (MOR) and 95% confidence intervals. MOR quantifies the variation between clusters by comparing two 'identical' persons from two randomly chosen, but different clusters. MOR expresses the heterogeneity on an odds ratio scale among clusters and represents the median increased risk. Consequently MOR can never be smaller than one. A cluster consists of all patients belonging to the same practice or network, respectively. In multilevel logistic regression analysis, MOR can be calculated for the network and practice level. In this paper, MOR implies that between two randomly chosen practices or networks, the risk of being diagnosed with a disease (i.e. diabetes mellitus) is x times higher in the randomly chosen network or practice with the highest occurrence rate compared to the risk of being diagnosed with that disease in the other randomly chosen network or practice with the a lowest occurrence rate [18,19].

We analysed the effect of socio-demographic characteristics in three steps. The first step consisted of analyzing the variation in an empty model (model 0), where no socio-demographic characteristics were taken into account. In the second step, the variation among networks and practices was adjusted for age and gender (model 1) and in the third step SES, level of urbanization and ethnicity were also considered (model 2). All analyses were carried out using SAS version 9.2.

## Results

### Socio-demographic characteristics

The total study population consisted of 487, 516 persons in 109 practices with a mean age of 38.5 years and almost fifty percent males (49.0%), see Table 1. The distribution of age and gender was comparable among GPRNs, the proportion of males ranged from 47.4 to 49.4 percent and the age differences among GPRNs varied in the age group under 20 years from 22.9 to 26.3 percent and the age group over 65 years from 11.4 to 17.5 percent. The distribution of SES, urbanization level and ethnicity was more diverse: the relative size of the low SES group ranged from 10.6 to 79.7 percent and some GPRNs operated almost exclusively in 'very urban' areas (highest rate 86.0%) while others operated mainly in 'rural' areas (highest rate 71.8%). Less than 0.5 percent of the population of CMR-N, RNUH-LEO, SMILE and Trans lived in neighbourhoods with 50% or more non western immigrants.

**Table 1:** Socio-demographic characteristics of General Practice Registration Networks

	*N *patient	*N *practice	% male	Age distribution			SES distribution			Urbanization			**NW immigrants**^**#**^			
				
				0 < 20	20 < 65	65+	Low	Medium	High	Very urban	Urban	Rural	0 < 10	10 < 50	50 < 70	≥70
CMR-N	10409	3	47.8	26.3	60.2	13.5	16.4	24.2	59.4	24.5	3.7	71.8	92.1	7.9	0.0	0.0

HAG-net-AMC	43930	7	47.4	24.7	63.9	11.4	79.7	13.0	7.3	86.0	0.4	13.5	13.7	9.8	60.3	16.3

LINH	327551	81	49.4	24.0	61.8	14.1	48.4	24.9	26.7	38.8	16.2	45.1	68.4	25.9	3.7	2.1

RNUH-LEO	34835	4	48.9	24.3	63.6	12.1	10.6	18.7	70.7	34.1	34.8	31.0	71.5	28.5	0.0	0.0

Smile	56799	9	48.5	22.9	59.6	17.5	70.1	7.4	22.5	66.7	22.0	11.4	17.0	83.0	0.0	0.0

Trans	13992	5	48.5	23.9	60.2	15.9	25.2	29.8	45.0	50.1	9.4	40.5	42.2	57.4	0.2	0.1

^#^NW immigrants = percentage of the population which live in neighbourhoods with almost no (0 < 10%), some (10 < 40%), many (40 < 70%) or most (≥70%) persons from non western origin

### Differences in morbidity estimations among GPRNs

Table 2 shows the included ICPC-1 codes of the diseases and disorders under study. The range of the incidence and prevalence rates among GPRNs is large (see table 2). For example, the estimated incidence rates of depression range from 4.4 to 14.2 per 1000 in 2007. We observed these relatively large differences in most diseases. This is also illustrated by the MOR. The results of model 0 illustrate the variations without adjusting for any socio-demographic covariates. If we consider the incidence rates of depression again, a MOR of **1.49 **(1.14-3.04) is shown among networks and **1.40 **(1.29-1.52) among practices. This implies that in two randomly chosen GPRNs, the risk of being diagnosed with depression is "on average" about 1.5 times higher in the GPRN with the highest incidence rate than in the GPRN with the lowest incidence rate.

**Table 2:** Variations in morbidity estimations of 13 diseases; incidence rates, prevalence rates and median odds ratios

Diseases	ICPC1 Codes	Incidence			Prevalence		
		
		Range of incidence (per 1000)	MOR (95%CI)		Range of prevalence (per 1000)	MOR (95%CI)	
					
			Network	Practice		Network	Practice
Gastrointestinal tract infection	D70, D73	10.4-28.2	**1.47**	**1.54**	n/a	n/a	n/a
					
			1.22-2.75	1.40-1.68		-	-

Diabetes mellitus	T90	3.3-4.6	1.00	**1.59**	30.9-57.2	**1.20**	**1.49**
					
			1.00-1.37	1.44-1.77		1.08-1.61	1.43-1.53

Depression	P03, P76	4.4-14.2	**1.49**	**1.40**	21.6-64.4	**1.58**	**1.70**
					
			1.14-3.04	1.29-1.52		1.27-3.05	1.61-1.79

Anxiety disorder	P01, P74	2.6-14.6	**1.71**	**1.52**	11.3-44.4	**1.64**	**1.73**
					
			1.22-4.26	1.39-1.67		1.29-3.32	1.65-1.82

Stroke	K89, K90	2.3-5.9	**1.38**	**1.47**	3.3-47.2	**1.85**	**1.78**
					
			1.10-2.49	1.33-1.65		1.42-4.28	1.74-1.88

CHD	K74, K75,	2.8-5.4	1.00	**1.71**	9.7-47.2	**1.78**	**1.86**
					
	K76		1.00-1.86	1.51-1.95		1.35-4.57	1.75-1.97

COPD	R91, R95	1.3-3.9	**1.40**	**1.54**	12.1-33.0	**1.35**	**1.65**
					
			1.09-2.87	1.39-1.73		1.14-2.14	1.57-1.73

Asthma	R96	2.9-6.2	**1.37**	**1.70**	29.2-60.0	**1.29**	**1.59**
					
			1.10-2.61	1.52-1.90		1.13-1.91	1.52-1.66

Urinary tract infection	U70, U71,	29.4-46.1	1.19	**1.35**	n/a	n/a	n/a
					
	U72		1.00-1.69	1.27-1.49		-	-

Dermatitis	S88, S87	16.9-57.0	**1.20**	**1.28**	27.9-161.2	**1.76**	**1.56**
					
			1.05-1.70	1.21-1.38		1.39-3.72	1.50-1.62

Osteoarthritis	L89, L90,	5.4-9.7	1.20	**1.51**	12.3-61.2	**1.86**	**1.62**
					
	L91		1.00-1.87	1.37-1.65		1.43-4.32	1.54-1.70

Neck and back problems	L01, L02,	42.3-78.9	**1.28**	**1.24**	29.8-302.5	**2.26**	**1.39**
					
	L03, L83,		1.13-1.94	1.19-1.33		1.63-6.44	1.35-1.43
	L84, L86						

Herpes zoster	S70	3.5-4.5	1.08	**1.23**	n/a	n/a	n/a
					
			1.00-1.34	1.11-1.36		-	-

**Bold **variation among network versus practices is significant (*p *< 0.05).

Statistical significant differences among GPRNs were found for most other diseases. There were some exceptions. The incidence rates of herpes zoster showed no significant differences among networks (MOR_network _= **1.08 **(1.00-1.34) *p*-value = 0.19), as did the incidence rates of diabetes mellitus, coronary heart disease, urinary tract infection and osteoarthritis.

In general, the amount of variation among practices is larger than among networks. This is visible in incidence rates of 10 out of 13 diseases and in prevalence rates of 6 out of 10 diseases. An evident example is diabetes mellitus, where the morbidity rate estimates of diabetes mellitus show relatively small differences among networks (incidence rates MOR_network _= **1.00 **(1.00-1.37) and prevalence rates MOR_networks _= **1.20 **(1.08-1.61) but the variations among practices are relatively large (incidence rates MOR_practice _= **1.59 **(1.44-1.77) and prevalence rates MOR_practice _= **1.49 **(1.43-1.53)).

Looking at differences among networks, relatively large differences (MOR > 1.40) were seen in the incidence rates of gastrointestinal tract infections, depression and anxiety disorders and the prevalence rates of depression, anxiety disorders, stroke, CHD, dermatitis, osteoarthritis and neck and back problems. Overall, the variation in incidence rates is smaller than the variation in prevalence rates among networks as well as among practices.

### Socio-demographic characteristics and differences in morbidity

The socio-demographic characteristics, age and gender contributed significantly to the morbidity estimates of all diseases (except gender in COPD). SES, ethnicity and urbanization level showed only a significant contribution to morbidity rate estimates for a part of the diseases under study (results not shown). Even though differences in the distribution of socio-demographic characteristics are apparent (Table 1) we observe only small changes in variation in morbidity estimates among GPRNs (Table 3 and 4). In most diseases the MOR seems to decrease after adjustment for population characteristics, although for some diseases, the MOR even increased. For example, the variations among GPRNs in incidence rates of depression with and without adjusting for socio-demographic characteristics, expressed in MOR, are **1.49 **(1.14-3.04) (no adjustments), **1.48 **(1.12-3.02) (age and gender) and **1.40 **(1.00-2.77) (adjusted for age, gender, SES, ethnicity, and urbanization level). Overall, accounting for socio-demographic characteristics did not explain the variation between GPRNs or practices, though in most diseases the confidence intervals of MOR became smaller.

**Table 3:** Variation in Incidence data in MOR adjusted for population characteristics

MOR (95%CI)	Model 0		Model 1		Model 2	
	
	-		Age, gender		Age, gender, SES, ethnicity and urbanization level	
	
	Network	Practice	Network	Practice	Network	Practice
Gastrointestinal tract infection	**1.47**	**1.54**	**1.45**	**1.51**	**1.40**	**1.47**
	
	1.22-2.75	1.40-1.68	1.21-2.66	1.38-1.64	1.17-2.50	1.36-1.61

Diabetes mellitus	1.00	**1.59**	1.00	**1.62**	1.00	**1.67**
	
	1.00-1.37	1.44-1.77	1.00-1.42	1.46-1.81	1.00-1.49	1.48-1.87

Depression	**1.49**	**1.40**	**1.48**	**1.41**	**1.40**	**1.41**
	
	1.14-3.04	1.29-1.52	1.12-3.02	1.31-1.54	1.00-2.77	1.30-1.55

Anxiety disorder	**1.71**	**1.52**	**1.70**	**1.51**	**1.63**	**1.52**
	
	1.22-4.26	1.39-1.67	1.21-4.21	1.38-1.66	1.18-3.86	1.39-1.67

Stroke	**1.38**	**1.47**	**1.27**	**1.40**	1.24	**1.38**
	
	1.10-2.49	1.33-1.65	1.03-2.08	1.30-1.56	1.00-2.02	1.29-1.40

CHD	1.00	**1.71**	1.00	**1.66**	-*	-*
	
	1.00-1.86	1.51-1.95	1.00-1.50	1.48-1.89		

COPD	**1.40**	**1.54**	**1.40**	**1.49**	**1.44**	**1.42**
	
	1.09-2.87	1.39-1.73	1.12-2.76	1.35-1.66	1.17-2.78	1.30-1.60

Asthma	**1.37**	**1.70**	**1.38**	**1.69**	**1.43**	**1.70**
	
	1.10-2.61	1.52-1.90	1.11-2.66	1.51-1.88	1.14-2.77	1.52-1.91

Urinary tract infection	1.19	**1.35**	**1.19**	**1.35**	**1.19**	**1.33**
	
	1.00-1.69	1.27-1.49	1.00-1.70	1.27-1.44	1.00-1.68	1.25-1.42

Dermatitis	**1.20**	**1.28**	**1.19**	**1.27**	**1.19**	**1.26**
	
	1.05-1.70	1.21-1.38	1.04-1.68	1.21-1.38	1.00-1.67	1.20-1.36

Osteoarthritis	1.20	**1.51**	-*	-*	-*	-*
	
	1.00-1.87	1.37-1.65				

Neck and back problems	**1.28**	**1.24**	**1.28**	**1.25**	**1.22**	**1.22**
	
	1.13-1.94	1.19-1.33	1.13-1.94	1.19-1.35	1.07-1.74	1.17-1.31

Herpes zoster	1.08	**1.23**	1.07	**1.17**	1.00	**1.20**
	
	1.00-1.34	1.11-1.36	1.00-1.29	1.00-1.31	1.00-1.26	1.06-1.34

**Bold **variation among network or variation among practices is significant (*p *< 0.05). *Analyses did not converge

**Table 4:** Variation in prevalence data in MOR adjusted for population characteristics

MOR (95%CI)	Model 0		Model 1		Model 2	
	
	-		Age, gender		Age, gender, SES, ethnicity and urbanization level	
	
	Network	Practice	Network	Practice	Network	Practice
Diabetes mellitus	**1.20**	**1.49**	**1.20**	**1.48**	**1.13**	**1.40**
	
	1.08-1.61	1.43-1.53	1.07-1.58	1.43-1.53	1.04-1.39	1.35-1.44

Depression	**1.58**	**1.70**	**1.58**	**1.72**	**1.53**	**1.70**
	
	1.27-3.05	1.61-1.79	1.27-3.06	1.64-1.81	1.24-2.84	1.61-1.78

Anxiety disorder	**1.64**	**1.73**	**1.64**	**1.74**	**1.53**	**1.73**
	
	1.29-3.32	1.65-1.82	1.29-3.30	1.65-1.83	1.22-2.87	1.64-1.83

Stroke	**1.85**	**1.78**	**1.81**	**1.73**	**1.82**	**1.72**
	
	1.42-4.28	1.74-1.88	1.40-4.08	1.70-1.83	1.40-4.20	1.69-1.83

CHD	**1.78**	**1.86**	**1.72**	**1.85**	**1.60**	**1.85**
	
	1.35-4.57	1.75-1.97	1.33-4.20	1.82-1.96	1.27-3.55	1.82-1.96

COPD	**1.35**	**1.65**	**1.33**	**1.63**	**1.30**	**1.60**
	
	1.14-2.14	1.57-1.73	1.13-2.08	1.61-1.70	1.11-1.95	1.58-1.68

Asthma	**1.29**	**1.59**	**1.30**	**1.58**	**1.24**	**1.61**
	
	1.13-1.91	1.52-1.66	1.13-1.93	1.52-1.65	1.08-1.73	1.54-1.68

Dermatitis	**1.76**	**1.56**	**1.76**	**1.56**	**1.79**	**1.58**
	
	1.39-3.72	1.50-1.62	1.39-3.73	1.50-1.63	1.40-3.89	1.51-1.64

Osteoarthritis	**1.86**	**1.62**	**1.89**	**1.59**	**1.88**	**1.58**
	
	1.43-4.32	1.54-1.70	1.45-4.47	1.57-1.66	1.44-4.37	1.56-1.66

Neck and back problems	**2.26**	**1.39**	**2.33**	**1.42**	**2.39**	**1.41**

	1.63-6.44	1.35-1.43	1.66-6.94	1.38-1.47	1.68-7.32	1.37-1.45

**Bold **variation among network or variation among practices is significant (*p *< 0.05)

## Discussion

Morbidity estimates can be derived from routine data collected in general practice. A setback for using these data for public health reporting is that morbidity estimates vary largely between different general practice registration networks (GPRNs). In this study we quantified these differences and studied the effect of socio-demographic characteristics of the population covered by the different GPRNs on the variations in 'episode based' morbidity data.

### Summary of main findings

There are large differences in morbidity rate estimates among GPRNs and these differences are more apparent for prevalence than for incidence rates. The risk of being diagnosed with a particular disease depends on the GPRN or general practice a patient belongs to. An exception is, for example, the incidence of diabetes mellitus which shows almost no variation. Differences in socio-demographic characteristics could not explain the variation in morbidity estimations among GPRNs.

### Differences among networks and among practices

Hardly any variations among GPRNs are observed in the incidence rates of diabetes mellitus, CHD, urinary tract infections, osteoarthritis and herpes zoster. Diabetes is a disease which can be clearly diagnosed. The same is true for urinary tract infection, osteoarthritis and herpes zoster, which are often painful and therefore patients are likely to seek medical care. For patients with CHD it is important to receive medical care and therefore these patients are nearly always known by the GP.

We expected differences among GPRNs and practices in morbidity estimates to be larger in diseases with more ambiguous diagnostic criteria [7]. In accordance with this expectation, large differences were seen in depression, anxiety disorders and gastrointestinal tract infections, where determination of these disorders depends highly on the presentation of the complaints to the GP.

Furthermore, large differences were expected in the prevalence rates of recurring diseases. Prevalence is influenced by the routine of closing episodes of diseases in the registration when the recurrence of the condition is over [3]. The large variations found in the prevalence rates of depression, dermatitis and neck and back problems might be explained by differences in these routines among GPRNs.

Interestingly, we expected large differences in diseases for which people receive little medical care, but this was only observed in the prevalence of osteoarthritis. We observed hardly any differences in incidence rates, which suggest that GPs see and diagnose relatively the same number of patients with osteoarthritis. This may also be true for neck and back problems. The large differences could be explained by different operational definitions and recording rules of prevalent cases in the different GPRNs. Defining a prevalent case in "episode based" data can be done in two ways: 1) a case is prevalent only when the patient has had at least one GP-contact for that disease in the year of interest or 2) all known cases with a previously recorded diagnoses for that disease, count as prevalent cases, irrespective whether a contact for that disease took place in the observation year. Osteoarthritis is a chronic disease, but since health care cannot always provide effective treatment patients do not necessarily contact their GP each year. These differences in recording rules may explain some of the variation in prevalence rates among GPRNs.

For most diseases differences are larger among practices than among GPRNs. This is apparent in the incidence rates of diabetes mellitus, even though diabetes mellitus has clear diagnostic criteria and results are adjusted for the socio-demographic characteristics of the patients. This can possibly be explained by coding qualities of practices within networks or differences in practice characteristics, but this was not investigated in this research. In this context it is also interesting to investigate the differences between strict and more interpretable recording rules on variation among practices.

### Influence of population characteristics

Although age and gender contribute significantly to the determination of morbidity, differences among GPRNs and among practices do not change after adjustment for these variables. This finding seems contradictive, but there are just small differences in age en gender distribution among GPRNs and therefore only small changes are possible.

The influence of SES, ethnicity and urbanization level is also limited, despite the large differences in distribution among GPRNs. We believe this to be the case due to little power, because of the small numbers of patients diagnosed with a disorder in comparison to the 'healthy' people. Furthermore if the socio-demographic characteristics significantly contribute to an improved morbidity estimation, as for example SES and ethnicity in back and neck problems (results not shown), this effect is too small to actually change MOR. The effects of socio-demographic characteristics are larger for prevalence rates and therefore we see more changes in variation among networks after adjustments. For example, SES and ethnicity significantly contribute to the prevalence of diabetes mellitus in patients (results not shown), and MOR declines from **1.20 **(1.08-1.61) to **1.13 **(1.04-1.39). Despite the small changes in variation after adjustment, differences among GPRNs and practices still remain large.

### Strengths and limitations of this study

To our knowledge, this is the first study to investigate the influence of socio-demographic characteristics on the variation of morbidity estimates among 'episode based' GPRNs. The distribution of age and gender in the different network populations corresponds reasonably well to the Dutch general population.

The differences in ethnicity and urbanization level are much larger among networks, which is caused by the fact that most networks operate regionally and the distribution of these characteristics is not equally distributed between regions in the Netherlands. Therefore we think adjusting for these characteristic is essential. Some GPRNs show an extreme distribution on some of the socio-demographic characteristics as, for example; more than 85% of the HAG-net-AMC population lives in very urban areas. Reanalysis without this GPRN did not lead to changes: some variations slightly increased, some decreased, and still hardly any changes were seen after adjusting for socio-demographic characteristics (results not shown).

To investigate the effect of socio-demographic characteristics, we adjusted for the differences in population composition among GPRNs. However, direct measures of SES and ethnicity were not available, and we had to rely on proxy measures. This may have led to an underestimation of the effects of SES and ethnicity because of these less accurate estimates. Overall, the relations found seem to be legitimate. For example, low SES was related to higher morbidity rates of diabetes mellitus and in COPD high SES was related to lower morbidity rates (results not shown) [20]. Although direct measures are more precise this could not explain that some variations even increase. Therefore we assume our conclusion, that socio-demographic characteristics do not explain differences among GPRNs, to be valid.

The differences in incidence estimations of herpes zoster among GPRNs are small and within the range seen in other research [12]. As the crude figures for herpes zoster show no significant variation among networks (MOR_network _= 1.08 (1.00-1.34)), we can conclude that the populations used are sufficient. It might even indicate a good recording quality of the GPRNs [12].

We only used 'episode based' data to rule out the differences due to different types of data. We have data of eight Dutch GPRNs, four networks only have 'episode based' morbidity data, two have 'problem based' data and two have both. Such a low number of GPRNs makes it impossible to include data type in the multilevel analyses. Other Dutch GPRNs did not want to participate or were not able to deliver their data on time. Dutch GPRNs differ from each other, but the distribution of the population characteristics in different GPRNs was broad and therefore we think considering other GPRNs would not have changed our conclusion.

## Conclusions

In a previous paper, we identified factors which may be responsible for the differences in morbidity among general practices and registration networks. Current research showed that one of the factors, the characteristics of the patient population, could not explain these differences. Understanding the differences between GPRNs and practices is a first step to come to the most valid and reliable estimate for the morbidity in the general population.

## Competing interests

The authors declare that they have no competing interests.

## Authors' contributions

CvdD, NH, FS and GW participated in the conceptual development, study design, the writing and editing of the article. CvdD was also responsible for data collection, data analysis and drafting of the manuscript. HCB supported the data analysis, verified analytic approaches and contributed to subsequent drafts. MvdA, MB, CvB, HJB, RV and MdW participated in data collection and contributed to subsequent drafts. All authors approved the final draft.

## Funding

This research was supported by a Strategic Research Program grant from the National Institute for Public Health and the Environment.

## Pre-publication history

The pre-publication history for this paper can be accessed here:

http://www.biomedcentral.com/1471-2458/11/887/prepub
